# Effects of Same-Sex Marriage Legalization for Sexual Minority Men in Taiwan: Findings From a Prospective Study

**DOI:** 10.3389/ijph.2022.1604489

**Published:** 2022-03-07

**Authors:** Yu-Te Huang, Zurong Liang

**Affiliations:** Department of Social Work and Social Administration, University of Hong Kong, Hong Kong, Hong Kong SAR, China

**Keywords:** panel data, Taiwan, effects, sexual minority men, same-sex marriage legalization

## Abstract

**Objective:** On May 24, 2019, same-sex marriage (SSM) was legalized in Taiwan. Increasing research in western countries has yielded longitudinal evidence about the psychosocial benefits of SSM for sexual minority individuals, but they have rarely included sexual minority-specific measures or considered participants’ relationship status. This study aimed to examine the short-term effects associated with the legalization of SSM for gay and bisexual men in Taiwan.

**Methods:** A panel sample of 731 gay and 132 bisexual men participated in baseline (May 2019) and follow-up (October 2020) online surveys to report their depressive symptoms, distal sexual minority stress, internalized homophobia, and outness status.

**Results:** The results demonstrated significant reductions in depressive symptoms and distal sexual minority stress along with increased rates of coming out to friends, family, and parents. These changes were similar for partnered and un-partnered individuals. Fixed-effect regression analysis indicated that the decline in distal sexual minority stress and internalized homophobia contributed to the decline in depressive symptoms.

**Conclusion:** This study preliminarily supports the positive effects of SSM in promoting sexual minority men’s mental health and disclosure in Taiwan.

## Introduction

On May 24, 2019, Taiwan has become the first in Asia to legalize same-sex marriage (SSM). The current legislation allows two same-sex adults to marry while their intents to become parents cannot be realized yet. Notably, the legalization of SSM in Taiwan does not suggest widespread societal support for same-sex relationships but rather is a result of years of political negotiations and advocacy [1]. A study has documented a marginal shift in the public attitudes towards SSM since the passage of legislation [2]. However, the legalization of SSM assumes vital implications for sexual minority individuals (i.e., lesbian, gay, and bisexual, LGB). On the one hand, legal SSM provides same-sex couples with an access to an array of rights and protections that were formerly exclusive to heterosexual married couples, such as tax exemption, social benefits, and hospital visitation rights [3]. These benefits are essential for the lives and relationships among married couples [4]. On the other hand, legalization of SSM represents an embodiment of the societal recognition of same-sex relationships [5]. One large-scale United States study found that both implicit and explicit anti-gay biases declined sharply following SSM legalization [6]. By affording institutional protections and mirroring societal acceptance, legalization of SSM has therefore been promoted as “a prescription for better health” for sexual minority communities [7].

The notion of structural stigma offers a theoretical underpinning for empirical research on the effects of SSM legalization. Structural stigma is defined “as societal-level conditions, cultural norms, and institutional policies that constrain the opportunities, resources, and well-being of the stigmatized” [8]. Viewed from this notion, the ban on marriage equality constitutes an institutional form of minority stress that has been linked to a myriad of adverse health and mental health outcomes among sexual minority individuals [9]. A review by Hatzenbuehler [10] identifies the direct and synergistic influences of structural stigma on stigma processes, such as driving concealment [11] and amplifying the adverse effects of rejection sensitivity [12]. Through various intra- and inter-personal processes, removal of structural stigma can benefit health and mental health among sexual minority individuals [13, 14].

The effects of SSM legalization have begun to be documented in longitudinal research. Hatzenbuehler, McLaughlin [15] extracted two-wave data from the National Epidemiologic Survey on Alcohol and Related Conditions and found that sexual minority individuals from the states that banned SSM during the 2004 and 2005 elections reported a rise in psychiatric disorders whereas their heterosexual peers did not. A quasi-experimental study showed that civil union legislation was associated with lower levels of stigma consciousness, perceived discrimination, depressive symptoms, and hazardous drinking among sexual minority women [16]. Raifman, Moscoe [17] obtained data from the Youth Risk Behavior Surveillance System to investigate the trends in suicide attempts during the legislation of SSM. Difference-in-differences analysis showed a 7% reduction in attempted suicide among youth who lived in the states that legalized SSM and such decline was more pronounced among sexual minority youth than their heterosexual peers.

Other longitudinal studies observed changes in service utilization as a proxy indicator of the effects of SSM. Hatzenbuehler, O'Cleirigh [18] drew on panel data from a health center in Massachusetts to examine the coincidence between the change in mental health consultations and expenditure and the legalization of SSM in 2003. They found significant reductions in visits to medical and mental health care services along with reduced expenses on mental health care when SSM was legalized. Another longitudinal analysis based on the Behavioral Risk Factor Surveillance System undertaken by Carpenter, Eppink [19] also found that access to legal marriage boosted marriage take-up for same-sex couples and improved healthcare access and utilization by sexual minority adult men.

Some studies are concerned with the impact of SSM legalization for those in a stable relationship. A series of studies by Ogolsky and his colleagues [20, 21] explored the mental health effects of federal recognition of SSM (i.e., the United States Supreme Court ruling in *Obergefell vs. Hodges*) on partnered individuals. These studies show that levels of stigma and stress were lower and family support and life satisfaction higher for individuals living in the states that legally recognized SSM [21]. Over the course of legalization, individuals in a same-sex relationship reported a decrease in the perception of stigma and an increase in family support. Although these findings have elucidated the effects of SSM legalization, the authors called for research to be extended to individuals who are not in a romantic relationship.

### Research Gaps

Despite this progress of empirical investigations, two methodological issues remain. First, there is limited analysis of data collected originally for assessing changes associated with SSM legalization [10]. Despite the ability to identify changes in outcomes in parallel with policy changes [16], a caveat in these studies involves the historical threat as researchers were not able to surely determine whether the observed outcomes were directly due to the SSM legislation or other confounding factors or parallel policy changes [16, 22]. A viable response to this methodological problem is to measure the outcomes specific to sexual minority individuals, such as exposure to distal sexual minority stress or disclosure status since they are intrinsic to the impact of legalization of SSM. A prospective design to assess LGB-specific outcomes in the same individuals is another solution by treating subjects as their own controls so that within-subject changes could be largely attributed to the policy changes [18].

Another gap pertains to the exploration of the effects of SSM for un-partnered sexual minority individuals [23] given that many studies [19, 24–26] only sampled partnered individuals. While certain benefits afforded by legalization of SSM are particularly relevant for LGB individuals in relationship, such as allowing them to form a family, increasing the sense of love, closeness, and commitment, and improving their relationships with family of origin [27], the reduction in discrimination and bias following the legalization [6] is supposed to benefit the entire LGB communities. However, studies have yielded inconsistent findings. A study with sexual minority women found that single participants perceived lower impact of SSM legalization on their personal lives and social climates than their partnered counterparts did [14]. In contrast, Hatzenbuehler, O'Cleirigh [18] found that changes in healthcare utilization following the legalization of SSM were not significantly different between partnered and un-partnered sexual minority men. Similarly, Everett et al. [16] found comparable health effects of legalizing same-sex civil unions for both partnered and un-partnered sexual minority women. Given the inconsistency of the study findings, we also accounted for the relationship status to determine whether SSM legalizations produced effects among wider sexual minority populations.

### The Current Study

To address these research gaps, this study employed a prospective, panel design to evaluate the short-term outcomes of SSM legalization in Taiwan. The study makes two empirical contributions. First, instead of capitalizing on pre-existing data, primary data were collected to delineate changes in mental health status and LGB-specific variables as functions of the legalization of SSM. The data gathered by this study are closely relevant to sexual minority individuals’ personal experiences and perceptions and therefore are more able to capture the direct implications of the marriage equality policy. Second, the sample comprised both partnered and un-partnered individuals, thus allowing us to ascertain whether the effects of SSM legalization varied by one’s relationship status. Notably, acknowledging the heterogeneity within sexual minority communities and the gender-based nature of experiences, we collected data from cisgender gay and bisexual men only.

## Methods

### Design and Sample

The study was prospective in nature, with baseline data collected 1 months prior to the enactment of SSM in Taiwan on May 24, 2019 and follow-up data gathered 1.5 years following the legislation. Similar with several previous studies [16, 18], we adopted this timeframe to assess the short-term changes associated with SSM legalization.

Data were derived from a community panel of self-identified gay and bisexual men living in Taiwan. We utilized Facebook advertising to recruit a baseline sample between May 11 and 23, 2019. A set of parameters were specified in the metrics to direct advertisements about the study to Facebook users who indicated in their profiles that they were: 1) Men, 2) aged ≥18 years, 3) residents of Taiwan, and 4) interested in lesbian, gay, bisexual, and transgender community–related issues and information. Interested users clicked the link embedded in the advertisement and were transferred to the online survey platform, *SurveyMonkey*, to read about the purposes of the study and eligibility criteria, which were: 1) Being assigned male at birth, 2) being aged 18 years or older, 3) self-identifying as gay or bisexual, and 4) residing in Taiwan. They pressed the button “I agree to participate” to give consent and spent approximately 12 min completing the survey. At the end of the survey, they were given the option of providing their contact details (e.g., email, mobile phone number, or communication mobile applications) to receive an e-coupon valued NT$100 as an honorarium.

Between September 14 and October 16, 2020, we sent invitations and information about the follow-up survey to respondents who had provided contact details. Respondents were informed of the purpose of the follow-up survey and ethical issues involved when considering whether to participate. After providing consent, respondents spent approximately 13 min completing the survey and were prompted to leave their contact information again to receive an honorarium. The data from the two waves were linked based on the respondents’ contact information.

In the baseline survey, 2,525 people visited the survey website, and 1,422 completed the questionnaire. Data cleaning was undertaken by inspecting respondents’ sociodemographic data, IP addresses, and contact information. Forty-one respondents were removed for: 1) being younger than 18 years (*n* = 3), 2) not identifying as gay or bisexual (*n* = 24), or 3) being repeat respondents (*n* = 14), thereby reducing the final sample of the baseline survey to 1,381. In the follow-up survey, only 952 responses were returned (attrition rate = 31.1%) of which 89 were discarded because: they were from an identical IP address as another respondent (*n* = 9); or had missing values in the full survey (*n* = 25), questions on the variables of interest (*n* = 30), or the age question (n = 5). Responses were also discarded if they had error messages (*n* = 1), were unmatched with the corresponding data in the baseline survey (*n* = 1), or not self-identified as bisexual or gay (*n* = 18). The final sample size for analysis was 863. The sociodemographic data of respondents from the follow-up survey are presented in Table 1.

**TABLE 1:** Sample characteristics stratified by relationship status (Effects of Same-Sex Marriage Legalization, Taiwan, 2019–2020).

	Baseline			Follow-up		
	Non-partnered (*n* = 412) *M* (*SD*)/*n* (%)	Partnered (*n* = 451) *M* (*SD*)/*n* (%)	*χ* ^*2*^/*t*	Non-partnered (*n* = 470) *M* (*SD*)/*n* (%)	Partnered (*n* = 393) *M* (*SD*)/*n* (%)	*χ* ^*2*^/*t*
Age	27.64 (5.78)	25.77 (5.74)	−4.77***	27.25 (5.85)	28.77 (5.64)	−3.85***
Sexual orientation						
Gay	352 (85.4)	355 (78.7)	6.57*	387 (82.3)	344 (87.5)	4.45*
Bisexual	60 (14.6)	96 (21.3)		83 (17.7)	49 (12.5)	
Employment status						
In employment	349 (84.7)	314 (69.6)	27.52***	372 (79.1)	344 (87.5)	10.64**
Not in employment	63 (15.3)	137 (30.4)		98 (20.9)	49 (12.5)	
Religious belief						
With belief	180 (43.7)	176 (39.0)	1.93	217 (46.2)	191 (48.6)	0.51
Without belief	232 (56.3)	275 (61.0)		253 (53.8)	202 (51.4)	
Educational attainment						
High school	19 (4.6)	40 (8.9)	11.34*	19 (4.0)	13 (3.3)	4.38
College or university	264 (64.1)	306 (67.9)		313 (66.6)	247 (62.8)	
Master’s degree	122 (29.6)	100 (22.2)		134 (28.5)	124 (31.6)	
Doctoral degree	7 (1.7)	5 (1.1)		4 (0.9)	9 (2.3)	
Monthly income						
No income	40 (9.7)	105 (23.3)	48.32***	69 (14.7)	25 (6.4)	37.12***
NT$0 – 20,000	75 (18.2)	116 (25.7)		92 (19.6)	57 (14.5)	
NT$20,001 – 40,000	147 (35.7)	127 (28.2)		166 (35.3)	125 (31.8)	
NT$40,001 – 60,000	100 (24.3)	77 (17.1)		87 (18.5)	130 (33.1)	
NT$60,001 – 80,000	32 (7.8)	17 (3.8)		34 (7.2)	30 (7.6)	
More than NT$80,001	18 (4.4)	9 (2.0)		22 (4.7)	26 (6.6)	

Note ***.*** *M*, mean; *SD*, standard deviation. ***p < 0.001, **p < 0.01, *p < 0.05.

### Data Collection

#### Measures

*Sociodemographic characteristics.* The survey included questions about respondents’ age, relationship status, sexual orientation, education level, monthly income, employment status, and religious belief.

*Depressive symptoms.* Respondents’ depressive symptoms were assessed by the Chinese version of the Patient Health Questionnaire-9 (PHQ-9), which has been validated with a community sample in Taiwan [28]. Respondents indicated the frequency of nine depressive symptoms during the past 2 weeks on a 4-point Likert scale (0 = never to 3 = nearly every day). A higher mean score represents a more severe level of depressive symptoms. The scale showed satisfactory internal consistency; Cronbach’s alphas were 0.88 in both the baseline and follow-up surveys.

*Distal sexual minority stress.* Respondents’ experience of distal sexual minority stress was measured by the Daily Sexual Minority Stressors Scale developed by Heron, Braitman [29]. Although the scale was originally developed for lesbian women, the questions were considered suitable for sexual minority men because they were adapted from existing measures that have been used for this population, including the Daily Heterosexist Experience Questionnaire [30]; the Heterosexist Harassment, Rejection, and Discrimination Scale [31]; and the Sexual Orientation Micro-aggression Scale [32]. The eight items assess perceptions and experiences of distal sexual minority stress (e.g., *I heard others make fun of, mock, or call sexual minority people names, such as fag or dyke; I was explicitly threatened with harm as a result of my sexual minority identity; I heard anti-LGB talk from family members*). For use with respondents in Taiwan, the scale was first translated into Chinese by a research assistant fluent in English and Mandarin and reviewed and revised by a research team involving several bilingual researchers. Responses to each item are on a 7-point Likert scale from 0 (not at all) to 6 (very much). A greater sum score indicates more frequent experiences of distal sexual minority stress. Cronbach’s alphas were 0.82 and 0.84 in the baseline and follow-up surveys, respectively.

*Internalized homophobia.* Negative attitudes and beliefs about sexual identity were measured using the Internalized Homophobia Scale [33] comprising eleven items regarding a respondent’s level of internalized homophobia. Sample items include *If possible, I would prefer to be a heterosexual* and *I am worried that my sexual orientation will disgrace my family.* Responses are measured on a 5-point Likert scale ranging from 1 (strongly disagree) to 5 (strongly agree). The scale was developed in a sample of gay Chinese men and has good internal consistency and construct validity [33]. In this study, Cronbach’s alphas were 0.82 in both the baseline and follow-up surveys.

*Outness.* Four questions were designed to assess the extents to which respondents have been “out” to other people. On a range from 1 (no one) to 5 (many), respondents answered the questions *How many of your friends know about your sexual orientation?* and *How many of your family members know about your sexual orientation?* In addition, two questions asked the respondent to indicate whether his father and mother were aware of his sexual orientation. Possible responses included yes, no, not sure, and not applicable (e.g., they had passed away).

### Data Analysis

We first described respondents’ sociodemographic characteristics stratified by their relationship status. Group differences were examined by chi-square tests and independent sample *t*-tests. Means and standard deviations of the continuous variables in the baseline and follow-up surveys were calculated separately and changes over time were examined by paired sample *t*-tests. The effect sizes of the t-tests were also computed. Designed to test paired nominal data, McNemar’s chi-square was used to assess the significance of the change in disclosure to mothers and fathers.

To examine the within-individual changes of the variables before and after the SSM legalization, we conducted repeated measures analysis of variance (ANOVA) to compare the mean differences across two time points. Because this analysis required individuals to have data at both time points, list-wise deletion was used to handle missing values. In the analysis, the independent variables were time as a 2-level within-subject (baseline and follow-up) measure and the relationship status in the follow-up survey as a 2-level between-subject factor (un-partnered and partnered). We tested the interaction effect of time and relationship status to examine whether changes over time were comparable between un-partnered and partnered respondents. These analyses were conducted using SPSS 24.0.

Provided with the panel data, we also used fixed-effect regression analysis to determine whether and how the changes in minority-specific factors, including distal sexual minority stress, internalized homophobia, and outness, contributed to the change in participants’ depressive symptoms. Fixed-effect analysis relies on within-person variation to estimate coefficients while automatically controlling for unobserved time-invariant factors when each individual serves as their own control [34]. Therefore, we only controlled for sociodemographic confounders that may change over time, including sexual orientation, relationship status, religious status, monthly income, and education attainment. To adjust for the time and contextual factor that may simultaneously affect people in Taiwan (e.g., COVID-19), we also created a dummy variable of time and incorporated it into each model. Each minority-specific factor was tested in separate models due to their inter-correlations. The Fixed-effect regressions were conducted using STATA 14.0.

## Results

The sample characteristics are presented in Table 1. The full sample was relatively young (mean age = 26.66, SD = 5.83 in baseline and mean age = 27.94, SD = 2.80 in follow-up) and predominately gay men (*n* = 707, 81.9% in baseline; *n* = 731, 84.7% in follow-up). Most respondents possessed a college/university degree or above (*n* = 804, 93.2% in baseline; *n* = 831, 98.5% in follow-up) and were in employment (*n* = 663, 76.8% in baseline; *n* = 716, 83% in follow-up). More than half (*n* = 507, 58.8% in baseline; *n* = *n* = 455, 52.7% in follow-up) had no religious belief. Respondents who had a monthly income above the median income (NT$40,000) account for 29.3% and 38.1% of the sample in baseline and follow-up, respectively. When stratified by relationship status, partnered respondents were slightly older, more likely to be gay, in employment, and have a higher monthly income in both baseline and follow-up data.

Descriptive statistics of the baseline and follow-up data and significance of changes over time are presented in Table 2. The paired t-tests indicate a significant reduction following SSM legalization in depressive symptoms (*t* = −4.81, *p* < 0.001, *d* = 0.17), distal sexual minority stress (*t* = −7.35, *p* < 0.001, *d* = 0.25), and an increase in disclosure to friends (*t* = 2.79, *p* < 0.01, *d* = 0.10) and family (*t* = 2.69, *p* < 0.01, *d* = 0.09). McMemar’s tests also showed a significant increase in the number of respondents coming out to their mother (χ^2^ = 5.82, *p* = 0.02) and father (χ^2^ = 9.14, *p* = 0.002).

**TABLE 2:** Descriptive statistics and changes over time (Effects of Same-Sex Marriage Legalization, Taiwan, 2019–2020).

	Baseline *M (SE)/n (%)*	Follow-up *M (SE)/n (%)*	95% *CI*	Cohen’s *d*	Significance *t/χ* ^*2*^
Depressive symptoms	6.37 (4.94)	5.64 (4.40)	−1.03, −0.43	0.17	−4.81***
Distal sexual minority stress	16.70 (7.38)	15.22 (7.56)	−1.88, −1.09	0.25	−7.35***
Internalized homophobia	30.80 (8.01)	30.44 (8.05)	−0.74, 0.01	0.07	−1.91
Disclosure to friends	3.85 (1.11)	3.92 (1.07)	0.02, 0.12	0.10	2.79**
Disclosure to family	2.50 (1.43)	2.58 (1.43)	0.02, 0.14	0.09	2.69**
Coming out to mother	275 (31.9)	295 (34.2)			5.82*
Coming out to father	171 (19.8)	196 (22.7)			9.14**

***Note.*** *SE*, standard error; *CI*, confidence interval. Paired-sample *t* tests were used to examine the mean difference between baseline and follow-up. McNemar’s chi-square tests were used to examine the differences between baseline and follow-up in the percentage of participants coming out to their mother or father. ***p < 0.001, **p < 0.01, *p < 0.05.

Table 3 shows the results of repeated measures ANOVA. These support the main effect of time on depressive symptoms (*F* = 21.65.10, *p* < 0.001), distal sexual minority stress (*F* = 51.60, *p* < 0.001), and disclosure to friends (F = 7.35, *p* < 0.01) and family (F = 7.65, *p* < 0.01). The interaction terms of time and relationship status were not significant, suggesting that the changes in depressive symptoms, distal sexual minority stress, and disclosure over time did not differ by relationship status.

**TABLE 3:** Within- individual changes and interaction effects (Effects of same-sex marriage legalization, Taiwan, 2019–2020).

	*n*	Baseline *M* (*SE*)	Follow-up *M* (*SE*)	Main effect of time	Time × relationship status interaction
Depressive symptoms					
Non-partnered	459	6.76 (5.14)	5.80 (4.55)	*F*_1_ = 21.65***	*F*_1_ = 2.81
Partnered	387	5.91 (4.66)	5.45 (4.20)		
Distal sexual minority stress					
Non-partnered	468	17.08 (7.57)	15.33 (7.69)	*F*_1_ = 51.60***	*F*_1_ = 2.16
Partnered	388	16.25 (7.12)	15.09 (7.40)		
Internalized homophobia					
Non-partnered	467	31.16 (8.01)	30.58 (8.08)	*F*_1_ = 3.19	*F*_1_ = 1.56
Partnered	390	30.37 (8.00)	30.27 (8.03)		
Disclosure to friends					
Non-partnered	470	3.78 (1.13)	3.87 (1.10)	*F*_1_ = 7.35**	*F*_1_ = 0.60
Partnered	393	3.93 (1.07)	3.98 (1.03)		
Disclosure to family					
Non-partnered	470	2.38 (1.43)	2.44 (1.43)	*F*_1_ = 7.65**	*F*_1_ = 0.93
Partnered	393	2.64 (1.42)	2.75 (1.42)		

Note. *M*, mean; *SE*, standard error. ***p < 0.001, **p < 0.0.

Table 4 presents the results of fixed-effect regression model. The significant coefficients of the time dummy variable across Model 1 to 6 indicated that controlling for the sociodemographic variables, there was a significant decline in participants’ depressive symptoms from baseline to follow-up. Model 1 and 2 show that the within-subject reductions in distal sexual minority stress (*B* = 0.05. *p* < 0.05) and internalized homophobia (*B* = 0.06. *p* < 0.05) contributed to the decrease in participants’ depressive symptoms.

**TABLE 4:** Fixed-effect regression model of the effect of minority-specific factors on the change in depressive symptoms (Effects of Same-Sex Marriage Legalization, Taiwan, 2019–2020).

	Model 1 *B (SE)*	Model 2 *B (SE)*	Model 3 *B (SE)*	Model 4 *B (SE)*	Model 5 *B (SE)*	Model 6 *B (SE)*
Time (*Ref.* = Baseline)	−0.65*** (0.17)	−0.72*** (0.17)	−0.72*** (0.17)	−0.72*** (0.17)	−0.73*** (0.17)	−71*** (0.17)
Distal sexual minority stress	0.05* (0.03)					
Internalized homophobia		0.06* (0.03)				
Disclosure to friends			0.01 (0.20)			
Disclosure to family				−0.05 (0.17)		
Coming out to mother					0.27 (0.57)	
Coming out to father						−0.28 (0.56)
Sexual orientation (*Ref.* = Bisexual)	0.31 (0.58)	0.35 (0.58)	0.22 (0.58)	0.23 (0.58)	0.21 (0.58)	0.22 (0.58)
Relationship status (*Ref.* = Not-partnered)	−0.45 (0.31)	−0.40 (0.31)	−0.41 (0.31)	−0.41 (0.31)	−0.42 (0.31)	−0.41 (0.31)
Employment status (*Ref.* = Not in employment)	−0.44 (0.43)	−0.37 (0.43)	−0.42 (0.43)	−0.42 (0.43)	−0.42 (0.43)	−0.42 (0.43)
Religious status (*Ref.* = Without belief)	−0.37 (0.39)	−0.35 (0.39)	−0.38 (0.39)	−0.37 (0.39)	−0.37 (0.38)	−0.38 (0.38)
Monthly income (*Ref.* = No income)	−0.08 (0.19)	−0.10 (0.19)	−0.06 (0.19)	−0.06 (0.19)	−0.06 (0.19)	−0.06 (0.19)
Education attainment (*Ref.* = High school)						
College or university	1.69* (0.78)	1.69* (0.78)	1.67* (0.78)	1.65* (0.78)	1.70* (0.78)	1.66* (0.78)
Master’s degree	1.70 (0.99)	1.70 (0.99)	1.65 (0.99)	1.63 (1.00)	1.69 (1.00)	1.64 (0.99)
Doctoral degree	2.42 (2.22)	1.48 (2.43)	2.14 (2.22)	2.13 (2.22)	2.17 (2.22)	2.12 (2.22)

Note. *B*, regression coefficient; *SE*, standard error. ***p < 0.001, **p < 0.01, *p < 0.05.

## Discussion

This is the first study to collect panel data to evaluate the short-term changes associated with SSM legalization among sexual minority men in Taiwan. Although pertinent research has grown in western countries that legalized SSM [14], this study extends the scope of evidence to an East Asian context where heterosexism still dominates various policy realms. Consistent with previous research [15, 16], this study found that when SSM is legalized, sexual minority individuals’ depressive symptoms reduce. Meanwhile, our respondents reported reduced exposure to distal sexual minority stress along with greater prevalence of disclosure to their family and friends following the legalization of SSM. There was also an increase in the number of respondents whose parents became aware of their sexual orientations. These changes might be due to the effect of SSM legalization in fostering a positive social climate through sending a public message to legitimize and normalize the intimate relationship between two same-sex adults. In line with the findings by Everett, Hatzenbuehler [16], this study demonstrated that SSM legalization also leads to a reduction in the perception of distal sexual minority stress, an effect that might stem from more open discussion about SSM, increased visibility of sexual minority communities, and expressions of support from other people. Notably, these significant changes were observed among both partnered and un-partnered respondents, suggesting that marriage equality policy has broad implications that are not limited to those actively considering marriage.

Surprisingly, the decline in internalized homophobia was not statistically significant. This may either be due to the overall low level of internalized homophobia at baseline or attributed to the endurance and complexity of this acquired attitude towards oneself. Given that internalized homophobia develops through an extensive period of time and from long-standing heterosexism, this cognitive schema appears less responsive to the short-term effect of SSM legalization and usually requires deliberate interventions and counselling to change [35]. Another noteworthy finding of this study is that after the legislation of SSM, sexual minority men became less reluctant to disclose their sexual identity to friends, families, and parents. This result has never been documented by previous research that relied on pre-existing data and also point to the culturally specific implication of SSM for the sociocultural context of this study where the cultural expectation to build a family and concern about “face” are routinely cited as barriers to parental acceptance of their LGB children [36]. It is likely that the legalization of SSM contributes to sexual minority men’s disclosure by opening a venue for them to form a family, albeit alternative, so as to fulfill social norms and expectations. This may account for participants’ stronger intention to be open about their identities.

### Study Limitations

Several limitations are noted. First, despite the longitudinal design, this study is limited in establishing causality of the effects of SSM legislation, which requires comparative samples of heterosexual individuals or LGB people not exposed to SSM legislation. Only through these comparisons are we able to ascertain whether the changes observed among LGB individuals are also attributable to other societal changes. Second, the representativeness of survey respondents is unknown because they were recruited through non-probability sampling and we could not determine how Facebook advertisement distributed the recruitment posts. The non-probability sampling procedure thus requires extreme caution in interpreting the result of inferential statistics. Third, as a common issue for Internet-based surveys [37], attrition bias could not be fully eliminated although no systematic patterns in drop out were found for the key variable (Appendix Table A1). Fourth, relative to objective measures, the self-reported measures on which the study relied could introduce self-report biases and measurement errors.

It should also be noted that the effects of legislation could be time-sensitive in that passage and implementation of the bill could produce differential impacts [16]. This consideration is also important for this study because the Supreme Court’s ruling on May 24, 2017 mandated the Legislative Yuan to either pass a new bill or amend the existing one to enact marriage equality within 2 years. Because the pre-legalization data were collected during this transitional period, participants might have been exposed to the anticipation effect of the policy shift. Meanwhile, the period preceding the enactment of SSM legislation was also marked by heated debate and a proliferation of opposing opinions and anti-gay comments. These social dynamics could have provoked emotions among members of LGB communities [38, 39]. The post-legalization data were collected during the COVID-19 pandemic that has imposed a collective influence on citizens’ mental health. All these events need to be considered in the interpretation of the findings while additional follow-up investigations will be necessary to examine the longer-term effects of SSM legalization.

### Conclusion

Advocacy of marriage equality is driven by values and evidence. While the ethos of equal rights aptly justifies the legalization of SSM, this study has generated explicit evidence for a wide range of psychosocial benefits afforded by SSM legalization to sexual minority men in Taiwan. The finding indicates that after the legalization of SSM, sexual minority men in Taiwan reported fewer depressive symptoms, perceived the environment as less hostile and exclusionary, and felt more willing to disclose their identities. These findings not only enrich knowledge about the effects of SSM policy but lend strong support for progression towards marriage equality.

## Appendix

**TABLE A1:** Differences in key variables between participants who completed both waves and who drop out from the follow-up survey (Effects of Same-Sex Marriage Legalization, Taiwan, 2019–2020).

Variables	Participation in both waves (*N* = 863) *M* (*SE*)/*n* (%)	Dropout from the follow-up (*N* = 559) *M* (*SE*)/*n* (%)	*χ* ^*2*^/*t* test
Depressive symptoms	15.47 (0.18)	15.95 (0.25)	*t* = 1.59^ns^
Life satisfaction	20.72 (0.21)	20.21 (0.27)	*t* = – 1.48^ns^
Distal sexual minority stress	24.70 (0.25)	24.93 (0.36)	*t* = 0.53^ns^
Internalized homophobia	30.80 (0.27)	30.41 (0.36)	*t* = – 0.88^ns^
Disclosure to friends	3.85 (0.04)	3.75 (0.05)	*t* = – 1.52^ns^
Disclosure to family	2.50 (0.05)	2.59 (0.06)	*t* = 1.19^ns^
Coming out to mother	275 (31.87%)	203 (36.31)	*χ* ^*2*^ = 3.01^ns^
Coming out to father	171 (19.81)	121 (21.65)	*χ* ^*2*^ = 0.70^ns^

Note. *M*, mean; *SE*, standard error; ns, non significance.
